# Indian Wheat Genomics Initiative for Harnessing the Potential of Wheat Germplasm Resources for Breeding Disease-Resistant, Nutrient-Dense, and Climate-Resilient Cultivars

**DOI:** 10.3389/fgene.2022.834366

**Published:** 2022-06-29

**Authors:** Sundeep Kumar, Sherry R. Jacob, Reyazul Rouf Mir, V. K. Vikas, Pawan Kulwal, Tilak Chandra, Satinder Kaur, Uttam Kumar, Suneel Kumar, Shailendra Sharma, Ravinder Singh, Sai Prasad, Anju Mahendru Singh, Amit Kumar Singh, Jyoti Kumari, M. S. Saharan, Subhash Chander Bhardwaj, Manoj Prasad, Sanjay Kalia, Kuldeep Singh

**Affiliations:** ^1^ Indian Council of Agricultural Research-National Bureau of Plant Genetic Resources, New Delhi, India; ^2^ Division of Genetics and Plant Breeding, Sher-e-Kashmir University of Agricultural Sciences and Technology of Kashmir (SKUAST-Kashmir), Jammu and Kashmir, India; ^3^ ICAR-Indian Agricultural Research Institute, New Delhi, India; ^4^ State Level Biotechnology Centre, Mahatma Phule Krishi Vidyapeeth, Rahuri, India; ^5^ School of Agricultural Biotechnology, Punjab Agricultural University, Ludhiana, India; ^6^ Borlaug Institute for South Asia, Ludhiana, India; ^7^ Department of Genetics and Plant Breeding, Chaudhary Charan Singh University, Meerut, Uttar Pradesh; ^8^ School of Biotechnology, Sher-e-Kashmir University of Agricultural Sciences and Technology of Jammu (SKUAST-Jammu), Jammu and Kashmir, India; ^9^ Indian Agriculture Research Institute Regional Research Station, Indore, India; ^10^ Division of Genetics, Indian Agricultural Research Institute, New Delhi, India; ^11^ Division of Plant Pathology, Indian Agricultural Research Institute, New Delhi, India; ^12^ CAR-Indian Institute of Wheat and Barley Research, Shimla, India; ^13^ Laboratory of Plant Virology, National Institute of Plant Genome Research, New Delhi, India; ^14^ Department of Biotechnology, Ministry of Science and Technology, New Delhi, India

**Keywords:** wheat, Indian wheat genomics initiative, genetic resources, genomics selection, gene bank, abiotic stress, biotic stress

## Abstract

Wheat is one of the major staple cereal food crops in India. However, most of the wheat-growing areas experience several biotic and abiotic stresses, resulting in poor quality grains and reduced yield. To ensure food security for the growing population in India, there is a compelling need to explore the untapped genetic diversity available in gene banks for the development of stress-resistant/tolerant cultivars. The improvement of any crop lies in exploring and harnessing the genetic diversity available in its genetic resources in the form of cultivated varieties, landraces, wild relatives, and related genera. A huge collection of wheat genetic resources is conserved in various gene banks across the globe. Molecular and phenotypic characterization followed by documentation of conserved genetic resources is a prerequisite for germplasm utilization in crop improvement. The National Genebank of India has an extensive and diverse collection of wheat germplasm, comprising Indian wheat landraces, primitive cultivars, breeding lines, and collection from other countries. The conserved germplasm can contribute immensely to the development of wheat cultivars with high levels of biotic and abiotic stress tolerance. Breeding wheat varieties that can give high yields under different stress environments has not made much headway due to high genotypes and environmental interaction, non-availability of truly resistant/tolerant germplasm, and non-availability of reliable markers linked with the QTL having a significant impact on resistance/tolerance. The development of new breeding technologies like genomic selection (GS), which takes into account the G × E interaction, will facilitate crop improvement through enhanced climate resilience, by combining biotic and abiotic stress resistance/tolerance and maximizing yield potential. In this review article, we have summarized different constraints being faced by Indian wheat-breeding programs, challenges in addressing biotic and abiotic stresses, and improving quality and nutrition. Efforts have been made to highlight the wealth of Indian wheat genetic resources available in our National Genebank and their evaluation for the identification of trait-specific germplasm. Promising genotypes to develop varieties of important targeted traits and the development of different genomics resources have also been highlighted.

## 1 Introduction

Wheat, a climate-sensitive crop, is grown on 31.76 million ha in India (ICAR-IIWBR, 2021). Most of the wheat-growing area faces several biotic and abiotic stresses and soil nutrient scarcity, resulting in poor quality grains and, finally, reduced yield (Pillay and Kumar, 2018; Grote et al., 2021). In the coming years, we will face a slew of challenges in ensuring food security for India’s millions of people (Dev and Sharma, 2010; Pandey et al., 2021). It is the need of the hour to explore the idle genetic diversity leading to the development of resilient and better-performing cultivars under challenging situations (Phogat et al., 2021). Breeding wheat varieties that can give high yields under different stress environments has not made much headway due to large genotype × environment interaction, non-availability of truly resistant germplasm, and non-availability of reliable markers linked with the QTL, having a significant impact on resistance to biotic and abiotic stresses and quality traits (Vishwakarma et al., 2015; Beres et al., 2020; Khakda et al., 2020; Xiong et al., 2021). Despite consistent efforts by breeders to utilize genetic resources in breeding programs which is reflected in the form of discussions, review articles, meetings, and presentations made on harnessing the advantage of genetic diversity present in conserved germplasm, there are only a few success stories till date where specific traits have been introgressed from traditional landraces or germplasm to elite breeding lines (Mascher et al., 2019; Sharma et al., 2021; Zeibig et al., 2021). One of several other reasons is the lack of concerted efforts to create a single platform for key people working on the harvesting of diversity at the national and international levels. Markedly, the dwarfing genes, which led to the Green Revolution, were introduced into wheat and rice breeding lines from East Asian landraces (Hedden, 2003). In the case of barley, the deployment of the *mlo* alleles, commonly found in Ethiopian barley landraces (Jorgensen, 1992), led to broad-spectrum resistance to powdery mildew. In 1967, Krull and Borlaug stated, “the problem at present is less, a lack of genetic variation, but rather of efficiency in identifying and incorporating it” (Pistorius, 1997). Currently, genebank managers, plant breeders, and geneticists have reached a consensus that there is an urgent need for the systematic evaluation of the evolutionary potential of large seed collections stored in cold rooms (Mascher et al., 2019; Singh et al., 2020; Zeibig et al., 2021). Collection and conservation of these genetic resources are the primary prerequisites for their access and use in crop improvement programs. Over the past several decades, concerted efforts have been made toward extensive collection and *ex situ* conservation of wheat germplasm accessions belonging to each gene pool category. Reportedly, around 800,000 wheat accessions are conserved across 80 different germplasm collections (Singh et al., 2007). These collections are proportionately represented in major global gene banks, the data for which can be accessed on the Genesys PGR platform. The Genesys database has information on 464,784 wheat accessions held in 40 global collections. The largest collection is that of CIMMYT, Mexico (150,178), followed by USDA-ARS (66,246), ICARDA (48,149), the Australian Grains Genebank (42,626), and the NI Vavilov Institute (35,314) (“https://www.genesys-pgr.org” accessed on 25 Jan 2022). Out of the total entries of wheat in the Genesys system, 7,535 accessions are of Indian origin, which are held in the Australian Grains Genebank (1,533), USDA-ARS (1,320), CIMMYT (1,315), John Innes Institute, United Kingdom (1,174), NI Vavilov Institute (1,154), and ICARDA (407) (Jacob et al., 2015; Gauchan and Joshi, 2019). A major proportion of these (1,879) are traditional cultivars or landraces, which constitute the high-priority genetic wealth in any crop (Azeez et al., 2018; Marone et al., 2021). Of the 7,535 accessions, 2,197 are declared as part of the multilateral system (MLS) of ITPGRFA and, hence, freely available for distribution to all signatories of the treaty from their respective holding institutes (Jacob et al., 2015; Vernooy, 2019). Even before the treaty regime, these Indian resources have made a significant contribution to global wheat programs, and the most significant examples are those of NP4 and Hard Red Calcutta (Singh S. K. et al., 2015). The NP4 and Hard Red Calcutta are prevalent in the pedigrees of several modern wheat varieties grown across the world. The National Genebank in India is the second-largest genebank in the world and has the fifth largest collection of wheat genetic resources, including several unique landraces and exotic ones (Tyagi, 2016; Phogat et al., 2021). Based on the *ex situ* germplasm collection size conserved in long-term storage, the Indian National Genebank has the second-largest collection in the world (a total of 459,885 accessions conserved in NGB, India, as on 31 March 2022), next only to the USDA Genebank (Tyagi et al., 2015). In the case of wheat, its *ex situ* collection in the National Genebank (34,000 accessions as on 31 March 2022) is the fifth-largest collection in the world (CIMMYT Genebank ranks first) (Jacob et al., 2015; Adhikari L. et al., 2022). These indigenous germplasm accessions are a valuable repository of economically important traits. The main goal of this review was to chart out a strategy for accelerating the use of this germplasm in the wheat-breeding program to address the various challenges in wheat production that in turn would minimize yield losses and maximize farmers’ income.

Most of the wheat-breeding programs across the world have relied more on limited sets of diverse genotypes. This has resulted in the narrowing of the genetic base of cultivated wheat and became a dominant production constraint. Therefore, an expansion in the genetic base of genotypes should be considered in the wheat-breeding program. This can be carried out in various ways: 1) use of plant genetic resources, including wild relatives and landraces; 2) germplasm-assisted breeding using advanced genomic tools; and 3) development of transgenic and use of modern techniques like gene editing. Several gene banks across the globe house a large number of diverse germplasm accessions of wheat. These germplasm accessions harbor many important genes not only for various biotic and abiotic stresses but also for nutritional qualities and yield traits. To harness the true potential of the vast wealth of accessions stored in the Indian National Genebank, the Department of Biotechnology (DBT), Govt. of India, has supported a mega project to dissect the available genetic resources for new trait discovery using genomics and phenomics approaches and their integration for improving climate resilience, productivity, and nutritional quality. In addition, it will also help in the identification of the novel QTL and the markers linked with these QTL for these traits. The presented reviews emphasized the dominant stresses limiting wheat production in India and its impact on global food security. The possible path of a second green revolution using preserved genetic resources in the Indian Genebank with prospects to make a necessary plan for the exigencies that may be arising due to various biotic and abiotic stresses, nutrient utilization, and sustainability was also discussed.

## 2 The Challenges: Improving Wheat Biotic and Abiotic Stress Resilience, Productivity, Quality, Nutrition, and Sustainability

Wheat is one of the key cereal crops not only in India but also in the world and is the primary grain consumed by humans around the world. It is a food source for around 35% of the world population, a major cereal crop, and the main contributor to the agricultural economy of India (Nagarajan, 2005; Joshi et al., 2007a) and needs to be systematically worked upon for sustenance and improvement. With a world population that is estimated to increase to nearly 10 billion by 2050, the demand for wheat would also increase at an annual rate of about 1.7% (Alexandratos and Bruinsma, 2012). On the other hand, wheat yield is growing at about 1% annually, hence, is not keeping pace with the increasing demand (Hatfield and Beres, 2019). Wheat production is being hampered by newly evolved, more aggressive pests and diseases, limited water resources, limited arable land, and rapidly changing climatic conditions (Beres et al., 2020). Wheat plays a substantial role in global food security and provides nutrition to a major part of the population in developing countries. Although with the breeding efforts made over the decades, several countries, including India, have attained self-sufficiency in wheat production, it is high time to think and plan for the future. This is important because the population is expected to grow at a higher rate than the dwindling land area for cultivation every year, pathogens are ever-evolving, and abiotic environments are constantly changing, all mingled with sudden outbreaks (Hussain, 2015; Hasanuzzaman et al., 2019). The first challenge to wheat productivity has been biotic stress, which is caused by an infinite, ever-evolving pathogen, and mining appropriate Indian wheat germplasm against such incidents is required.

### 2.1 Biotic Stresses

The production and productivity of wheat crop is hampered by various diseases including, rusts (leaf rust, stem rust, and stripe rust), powdery mildew, spot blotch, Karnal bunt, and *Fusarium* head blight (Singh and Rajaram, 2002; Hussain 2015; Vikas et al., 2020; Roy et al., 2021). Although, continuous efforts have been made to develop disease-resistant wheat varieties for several devastating diseases, it is also true that knocking down of the resistance genes against these diseases happens simultaneously (Ahirwar et al., 2018; Kumar et al., 2018). This is where the emphasis is needed because resistance breeding programs have frequently relied on single major genes, and there is large-scale cultivation of genotypes with almost identical resistance (Vikas et al., 2020; Roy et al., 2021). Moreover, due to directional selection, the genetic base has become narrow, leading to a monoculture (Bourke et al., 2021). This is going to be a very serious threat to wheat production in the coming decades. There is a need for the identification and stacking of multiple resistance genes for a particular disease in a single genotype so that the duration of resistance can be increased. Similarly, the combination of resistance genes for multiple diseases can prove very effective in tackling any kind of disease epidemic. There have been frequent breakdowns of deployed resistance against major diseases such as rusts, powdery mildew, and spot blotch. This suggests a need for continuous effort to search for novel sources of durable resistance against the emerging virulent races of wheat rusts in available wheat germplasm.

#### 2.1.1 Rusts

Rusts are still a significant biotic stress in wheat. Various researchers describe a capitulate loss of 10–100% in wheat due to rust diseases, which depends on the genotype of the cultivar, whether resistant or susceptible, inceptive infection time, rate of pathogenesis, duration of the disease, virulence factor, and the environment (Singh G. et al., 2017; Bhardwaj et al., 2019). Rust losses can vary from one year to the next and from region to region (Sawhney, 1995). The first stem rust epidemic was reported in 1786 in Madhya Pradesh, a major wheat-growing state in India (Nagarajan and Joshi, 1985), while we have experienced the continuous incidence of stripe rust in the northern part of India (Gupta and Kant, 2012; Vaibhav et al., 2017). *Puccinia graminis* f. sp. *tritici*, a causative agent of stem rust, resulted in up to 100% yield loss (Leonard and Szabo, 2005). The instantaneous emergence of a novel race of stem rust in Africa called *Ug99* spread to the Middle East, Iran, and other countries, making it a serious concern for global wheat productivity (Singh et al., 2008; Singh R. P. et al., 2015). This race was compatible enough to break down the *Sr31* gene, which has been widely used by breeders against stem rust to have a sufficient level of resistance for over two decades (Pretorius et al., 2000). This pathogen has been rapidly evolving since 1999, resulting in thirteen diverse variants under one lineage (RustTracker.org, 2019). In India, stem rust threatens approximately seven million ha of wheat-growing area (Bhardwaj et al., 2019). Similarly, stripe rust caused by *P*. *striiformis* Eriks. is dominating the northern provinces of India (Bhardwaj et al., 2019) and takes a heavy toll by reducing annual yields by about 30–50%. The wheat variety PBW343, which was a ruling variety in the North Western Plains Zone of India, has succumbed to stripe rust (Singh R. P. et al., 2017; Bhardwaj et al., 2019). The gross capital loss expected due to leaf rust (Caused by *P. tririciana*) pathogen varies and may be up to 60% under severe conditions (McIntosh, 1998).

Breeding for disease resistance is the most economic and imperishable component of integrated crop disease management (Vasistha et al., 2017; Kumar S. et al., 2015). Approximately, 83, 80, and 61 stripes, leaf, and stem rust resistance genes, respectively, have been curated and cataloged in wheat (McIntosh, 2020). However, the prompt evolution of novel virulent races makes most of the resistance genes ineffective. Unfortunately, the majority of the Indian wheat cultivars lack resistance to stripe rust and their tolerance has been fleeting even though they were evaluated as possessing an adequate level of resistance before being released to farmers. This posed a need for a durable and sustainable solution. In wheat, genetic resistance to rust pathogens can be categorized as follows: 1) all-stage resistance and race-specific or seedling resistance conferred by major genes (Chen, 2013); 2) race-specific adult plant resistance (APR); and 3) partial resistance and slow-rusting or non-race-specific adult plant resistance conferred by minor genes (Johnson and Law, 1973; Das et al., 1992). If resistance genes are used alone, there is a danger of the outbreak of a disease, but if several genes are combined into a single genotype (gene pyramiding), the duration of efficient resistance can be increased. The combination of minor genes with major disease resistance genes has been found to be effective and can attain durable resistance.

#### 2.1.2 Karnal Bunt

*Tilletia indica* is the causative agent of Karnal bunt, a disease with the greatest impact on the grain and food industry. The disease not only causes yield loss but also adversely affects grain quality due to infested kernels (Fuentes-Dávila and Rajaram, 1994). Grain infested with Karnal bunt attracts quarantine regulations that restrict infested seeds' transboundary movement. It was first reported in Karnal, India (Mitra, 1931), and was characterized as a minor disease till 1968. The disease was further ascertained in innumerable other regions throughout Northern and Central India. Later on, the disease was observed in several other countries, such as Nepal, Afghanistan, Iran, Iraq, Pakistan, Mexico, South Africa, and the United States (Rush et al., 2005). The pathogen infects wheat at the heading stage before seed formation; hence, the symptom is manifested only when the grains are matured in the ear heads. Traditional use of genetic resistance could be the best solution to manage disease severity. Although a huge collection of resistance sources was retrieved from diverse adapted zones, very few of them have been studied for detailed genetic analyses and used in the breeding program. Development of genetic markers, mapping of resistance genes, and characterization of new resistance loci can help to develop improved cultivars using germplasm (Brar et al., 2018).

#### 2.1.3 *Fusarium* Head Blight (FHB)

FHB, or head scab, is caused by different *Fusarium* species where *F. graminearum* and *F. culmorum* are considered dangerous due to the contamination of the grains by mycotoxins like deoxynivalenol (DON), nivalenol (NIV), and zearalenone (ZON). Yield losses occur due to shriveled grain, low test weight, and failure of seed formation. Mycotoxin accumulation is a major concern from an international trade perspective. Mycotoxins, especially DON and its acetylated forms (3-ADON and 15-ADON), make grain unsuited for food or feed upon accumulation (Brar et al., 2019). Although the import or export of FHB-infested wheat grain across international boundaries is allowed by defining a certain threshold, many beverage and food industries have self-imposed regulations (McMullen et al., 2012). Like with other diseases, the adoption of resistant cultivars is the most effective and convenient way to control this disease (Steiner et al., 2017). The complex genetics of FHB resistance makes it difficult to dissect desired resistance because it is under multigene control and associated with genotype × environment interactions. The classic example includes *Fhb1* derived from a Chinese variety, Sumai 3 that provides resistance against FHB was popularized by various breeding programs (Lv et al., 2014). However, *Fhb2* from Sumai 3 (Lu et al., 2010) and *Fhb7* (Guo et al., 2015; Wang et al., 2020) from *Thinopyrum ponticum* have also been used in the resistant breeding program. Meanwhile, pyramiding resistance genes into susceptible cultivars remains a formidable challenge because major sources of resistance genes (such as Sumai 3 and Wangshuibai) are associated with undesirable agronomic traits (Dvorjak, 2014; Li et al., 2016). In the Indian context, it causes notable yield loss if rain coincides with anthesis, which is prevalent in Punjab, Himachal Pradesh, Uttarakhand, and hilly areas of Tamil Nadu. There is a dire need for incorporating resistance against FHB in Indian cultivars, keeping in view the importance of increasing exports of Indian wheat. Germplasm could be the ideal source of resistance for sustainable approaches against such cataclysmic diseases.

#### 2.1.4 Spot Blotch

Spot blotch (SB), a destructive leaf disease of wheat caused by *Cochliobolus sativus* (anamorph: *Bipolaris sorokiniana*), is considered an economically important disease prevalent worldwide. This disease could result in as high as 70% yield losses under severe epidemic conditions (Ayana et al., 2018). The disease-favoring climate is more prevalent in South Asian and American countries, where warm and humid conditions persist throughout the wheat cropping season (Saari, 1998; Joshi et al., 2007b; Gupta P. K. et al., 2018). From the Indian perspective, the eastern parts are the main epidemic zones, from where it is spread into the cooler traditional rice–wheat areas like the North West Plain Zone (NWPZ) (Villareal et al., 1995; Joshi et al., 2007a). The resistance level in high-yielding wheat genotypes is unsatisfactory and needs to be improved remarkably, mainly in the humid regions of South Asia (Sharma and Duveiller, 2006; Joshi et al., 2007b). Complex quantitative inheritance of SB resistance in wheat has slowed the progress in breeding for SB resistance (Kumar S. et al., 2015). Crucial findings using both bi-parental mapping populations and association mapping panels have accessed assorted SB resistance QTL on all chromosomes except 1D, 3B, 3D, 4A, 4B, 4D, 5D, and 6A (Lu et al., 2016; Gupta P. K. et al., 2018). Nevertheless, only three prime QTLs were assigned, *Sb1* on 7D (Lillemo et al., 2013), *Sb2* on 5B (Kumar S. et al., 2015), and *Sb3* on 3B (Lu et al., 2016). Accessing novel resistance genes by exploiting wheat germplasm could be vital against such a ruinous disease.

#### 2.1.5 Powdery Mildew

Another cataclysmic disease caused by the biotrophic fungus *Blumeria graminis* (DC) E.U. Speer f. sp. *tritici* Em. Marchal (Syn. *Erysiphe graminis* DC f. sp. *tritici,* Em. Marchal) is powdery mildew (PM) of wheat, a foliar disease of universal occurrence resulting in dreadful yield loss (Mwale et al., 2017). Its severity usually climaxes in areas with high precipitation and a maritime-like climate (Bennett, 1984). However, it has gained importance in other regions due to the application of a higher dose of nitrogenous fertilizer and the cultivation of modern semi-dwarf wheat genotypes (Wang et al., 2005; Morgounov et al., 2012). As far as yield losses are concerned, they range from 15 to 40% depending upon the varieties and climatic conditions. Earlier, this disease was confined to the North Hill Zone (NHZ) of India, but now it is also spreading toward the North-Western Plains Zone (NWPZ) of India due to climate change, which has led to the development of new races. The gene with the highest level of resistance was studied on the wheat-rye translocation (1B/1R) fragment that originated from the cultivar Veery. This cultivar was substantially used to develop many PM-resistant cultivars around the globe (Friebe and Heun, 1989), including India (Vikas et al., 2020). Also, 68 loci providing resistance against wheat PM have been mapped to various wheat chromosomes (McIntosh, 2020). Moreover, the finding of several PM resistance genes, research should be carried out on finding novel resources to unravel genes, alleles, and SNPs because of the breakdown of truly race-specific resistance genes due to the emergence of new pathotypes or races (Hsam et al., 2003). Hence, it is necessary to explore adaptive wheat germplasm to identify refreshed resistance genes and use them against rapidly evolving pathogens.

#### 2.1.6 Other Pathogen/Pests

**FIGURE 1:**
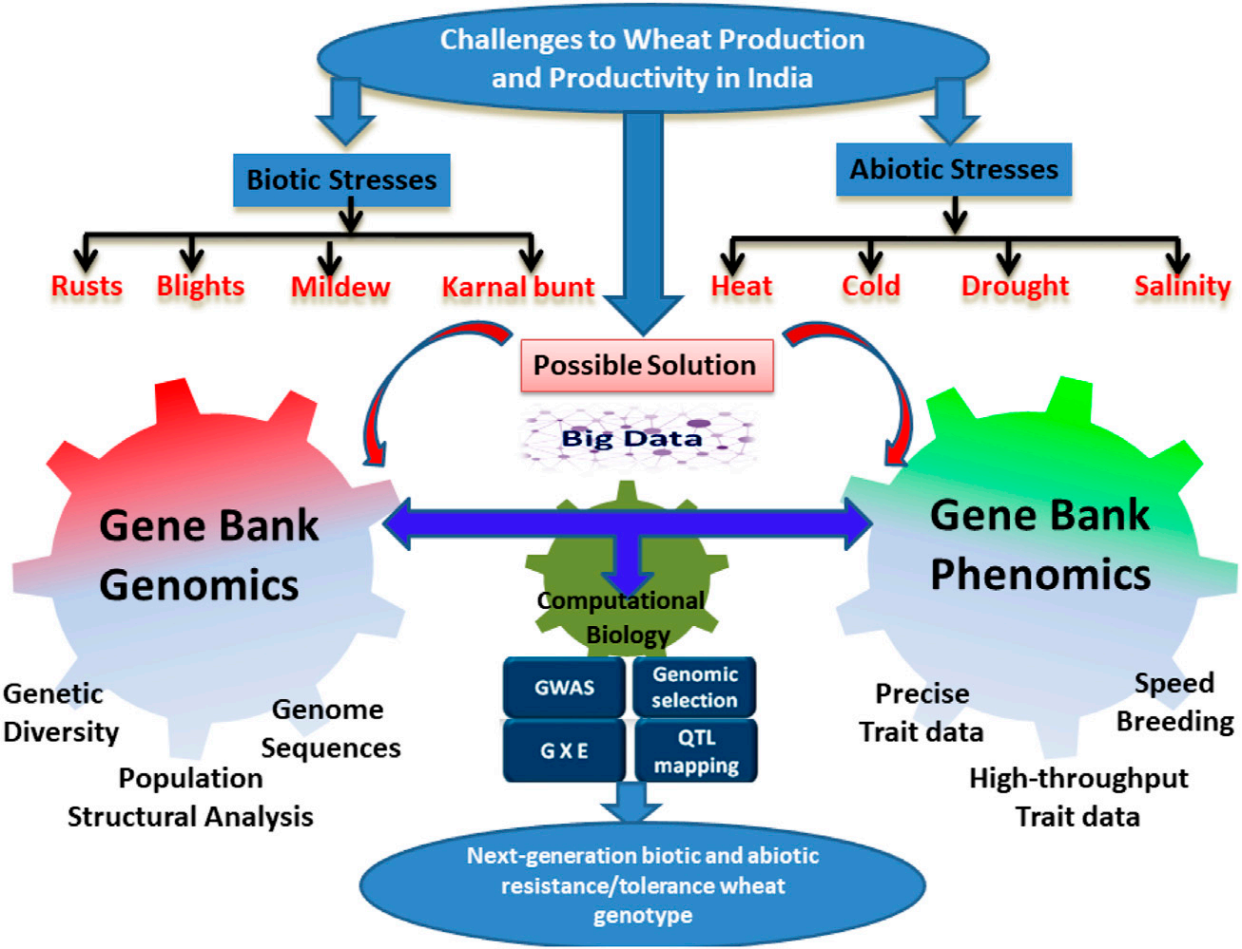
Challenges faced by Indian wheat-breeding programs and the possible solution. The figures show different biotic and abiotic stresses limiting wheat production and productivity in India. The figures also explain how genomic interventions and gene bank phenomics could be used in development of next-generation wheat varieties with enhanced biotic and abiotic stresses.

The yield losses due to insect pests have increased in the post-Green Revolution era (Dhaliwal et al., 2010). Unlike biotic stress resistance, the resistance gene had a very minor contribution in protecting wheat against insect pests because of the high impact of environmental conditions like temperature and light on the survival and behavior of the insects (Alford et al., 2014). Thus, a more concerted effort and methodology are required to identify and recruit the most effective insect pest resistance genes. Amongst the different pests, aphids, wheat weevil, wheat midge, termites, Hessian fly, armyworm, and cereal cyst nematode (CCN) are important arthropods feeding on wheat. CCN is becoming a serious threat to wheat production in several states of India (Singh and Kaur, 2015; Smiley et al., 2017). There is a higher perception of the CCN threat due to the fact that the identified genes confer a limited level of resistance to specific CCN pathotypes. Since the molecular mechanism of known genes is not known, it is essential to identify new genes and understand their interactions and functions in conferring resistance to CCN. Figure 1 basically explains the challenges faced by biotic and abiotic stresses in wheat production for the Indian wheat-breeding program which in turn accelerates or develops genebank genomic selection models using a combination of genebank genomics, genetic diversity, population structural analysis, and genomic sequences with phenomics, precise trait data, high throughput trait data, and speed breeding which is effective for speeding up the use of wheat germplasm lines coupled with breeding to enhance the process of variety development club with the genomic selection.

### 2.2 Abiotic Stress

Abiotic stresses are equally important which limit wheat production worldwide. Among abiotic stresses, salt, drought, and terminal heat stress are the three utmost constraints for successful wheat production in most parts of India. Climate change has been shown to have a high impact on wheat yield due to rising temperatures and water scarcity in India and other wheat-growing regions of the world. Wheat grain filling is suppressed at a temperature above 30°C because of reduced starch synthase activity (Jenner, 1994). Short-term extreme increases in temperature of 5–10°C can have quite catastrophic effects on yield, as an increase in the ethylene signal after heat spikes has been shown to lead directly to grain abortion (Hays et al., 2007). Moreover, it is estimated that a 1°C increase in temperature can result in a 10% decrease in wheat productivity in low-altitude countries (Lobell et al., 2011). Heat stress at the terminal stage of the wheat crop is a crucial abiotic stress that restricts plant growth and the accumulation of starch, which in turn causes yield unpredictability in many wheat-growing parts of the world (Gupta et al., 2012). Different reports have predicted that the average global temperatures will increase in the coming years (Malhi et al., 2021). With an estimated rise in global temperatures of up to 1.5°C by the year 2030 and a 1.8–4°C rise by the end of the century, the challenges facing wheat production are enormous and need to be tackled immediately. In addition to this, the pattern of diurnal and nocturnal temperatures has also started changing, which is resulting in warmer nights (Gupta et al., 2012). Heat stress, particularly at the terminal stage of wheat, is the major limiting factor for plant productivity and is a major cause of yield instability in many parts of the world. Changes in temperature patterns accompanied by unpredictable rainfall patterns are also affecting crop productivity in several countries, including India (Singh G. et al., 2018). The threat perceptions due to the impact of global climate change on agriculture are going to be huge in the coming decades. There is a complex genetic basis for most of the improved traits in wheat related to water-deficient and heat-stress conditions (Sallam et al., 2019). This is because each of these traits is polygenic and each gene has a small effect. Improvement of polygenic traits is itself a difficult task (Ranjan et al., 2021). The genetics behind abiotic stresses is more complex as compared to that of biotic stress. Although many studies have been conducted to elucidate the genetics of these traits, only limited success has been achieved in utilizing the vast wealth of data in the crop improvement programs. Global warming is severely affecting weather patterns, resulting in extremes of temperature, drought, frequent frost, and snowfall in high altitudes (IPCC. Climate Change, 2013). In the last few years, droughts and heatwaves have become frequent in a large part of India, posing a serious threat to future wheat production. In an estimate, the average yield loss of wheat in India due to a 1°C rise in temperature is reported to be 9.1 ± 5.4%, while the global yield loss triggered by the same is projected to be 5.5%, accounting for an aggregate loss of 35 M tons (Wang et al., 2018). This calls for the deployment of varieties that can withstand heat stress during the anthesis and seed setting stages. Furthermore, the accelerated use of wheat germplasm in a sustainable and planned manner is a viable option for addressing biotic, abiotic, and malnutrition threats.

In the last few decades, due to drastic changes in climatic conditions, most of the world faced low water accessibility, especially in South Asia and Africa. Among all the abiotic stresses, drought and terminal heat stress are the major limitations to food production worldwide, including India. Hence, developing genotypes that hold terminal heat tolerance is one of the crucial precedents of wheat improvement programs in India. The continuous shrinking of water resources around the world has further compounded problems, in addition to thermal stress, leading to reduced production and productivity (World Meteorological Organization, 1997), and there is a need for additional sustainable approaches to increasing productivity on restricted land, which will prevent the detrition of biodiversity (Pretty and Bharucha, 2014). Climate change is predicted to have a high impact due to rising temperatures and water scarcity in the densely populated regions of India. In many of the global wheat-growing areas, drought and terminal heat stress cause maximum damage.

#### 2.2.1 Terminal Heat Stress

Around nine million ha of wheat in the subtropical or tropical zone (Lillemo et al., 2005) are heat stressed in countries including India, Bangladesh, Uganda, Nigeria, Sudan, and Egypt that have traditions of cultivating wheat since long ago (Abdelmageed et al., 2019). An estimate suggests that India’s 13.5 million ha of wheat cultivated land comes under a heat-stressed zone (Joshi et al., 2007a). Terminal heat stress is one of the measures of sudden remarkable enhancement in temperature during the grain filling stage till maturity. The mean temperature above 31°C during caryopsis ripening in wheat comes under the influence of terminal heat (Kumari et al., 2015; Dubey et al., 2020). Due to climatic fluctuation, the commencement of early summer than normal and late sowing of wheat due to a mixed cropping system are the possible factor for terminal heat stress in wheat (Gupta et al., 2012). Terminal heat stress causes severe damage to wheat, which alters its physiology and grain filling mechanism. Intense high-temperature waves are likely to become more damaging if the current trends continue and future predictions about global warming hold true. Notably, it significantly impacts starch synthesis and accumulation which is a measure of grain filling rate and gross productivity declined by the sudden outbreak of heatwave during caryopsis development (Jenner, 1994; Kumar et al., 2016c). Furthermore, current approaches for crop management utilize the application of irrigation water, which can reduce heat stress on plants (Badaruddin et al., 1999) but is not feasible for large areas. To date, our limited understanding of the complex interaction of cellular/molecular mechanisms with whole-plant adaptation has restricted deterministic approaches to breeding for heat tolerance (Reynolds et al., 2021). Germplasm could be a reliable source of gene or QTL for heat tolerance, especially at the ripening stage and seed maturation. Additionally, it could be managed by the introduction of trait-like late maturity genotype, stay green-harboring germplasm, and their wider use for adaptability against the current scenario.

#### 2.2.2 Drought Stress

Drought is the second most serious abiotic stress limiting wheat production in different parts of the world and occurs with varying frequencies (Boyer, 1982; Chaves et al., 2003). Drought affects wheat crops more frequently in tropical and subtropical regions, where most of the developing countries are situated. Around 17% of the cultivated wheat areas worldwide were affected by drought during the period of 1980–2006 (Dai, 2013). In India, 29% of the total cultivable area faces drought conditions, of which 10% is under severe drought (Anonymous, 2003). This has caused an estimated 20–30% reduction in total wheat yield in stressed areas. Reduced bioavailability of water across the heatwave at the terminal growth phase of the spike is negatively correlated with productivity. Basically, they both occur at the same time, and their additive effect causes aborted grain filling (Sattar et al., 2020). Drought stresses impact on their own or in combination that significantly affect several agronomical features like heading days, the height of the plant, numbers of tiller per plant, and length and occupancy of the spike. However, an indirect correlation was suggested in terms of expressed results of GWAS or mapped QTL possibly due to a paradoxical association between traits and genetic loci (Tahmasebi et al., 2016; Abou-Elwafa and Shehzad, 2021). Therefore, a significant effort will be required, including molecular tools to breed superior drought-tolerant varieties. Whole-genome sequencing for each genotype was not possible earlier, but the commencement of high-throughput sequencing technology makes it accessible for extreme landrace and exotic lines (Khadka et al., 2020). The number of genes contributing directly or indirectly to drought tolerance relies on the associated traits' magnitude and proximity of the genes associated with the markers. Genome-wide association studies (GWAS) or QTL mapping could be used to identify genes, involved in drought tolerance in unexplored germplasm, which could then be used to improve crop drought tolerance (Zeng et al., 2014; Sukumaran et al., 2018). Furthermore, advanced breeding programs for crop improvement are assisted by genomic selection and gene editing for improving drought tolerance in wheat (Singh S. K. et al., 2015).

#### 2.2.3 Salinity

Among abiotic stresses, increased soil salinity and sodicity pose a challenge to agriculture. The high salt concentrations of the soil can be attributed to the poor land and water management practices as well as the lack of soil reclamation processes in many parts of the world. In India, approximately 8.6 mha of the cultivated land is affected by soil salinity. Furthermore, the areas under salinity are expanding each year due to low precipitation, mixing with the coastline, saline water irrigation, high surface evaporation, and poor cultural practices (Jamil et al., 2011). It has been extrapolated that about 50% of the cultivated land area may be impregnated with salt by the mid of twenty-first century (Mahajan and Tuteja, 2005; Sharma et al., 2012). Salinity tolerance could be accessed by using conventional (El-Hendawy et al., 2005) to modern spectral imaging techniques (Moghimi et al., 2018). Although, most of the affected parameters were known for salt tolerance which limits productivity, inadequate large-scale phenotyping could be a possible factor for a significant outcome (El-Hendawy et al., 2005; Rosenqvist et al., 2019). Finding well-studied genes/transcription factors from wheat germplasm like AVP1, NHX2, DREB, and SHN1 and their associated marker (Díaz De León et al., 2010; Goyal et al., 2016; Singh A. K. et al., 2018; Kumar et al., 2020c) for the utilization for tolerance breeding could be a sustainable approach for generating salt-tolerant wheat genotypes (Choudhary et al., 2021). Hence, there is a compelling need to develop salt-tolerant wheat varieties. Although in the context of salt tolerance germplasm utilization, there is little progress yet, notably germplasm which harbors extreme salt-tolerant genes could be rescued for generation of pre-breeding lines for crop breeding which could stand against the high saline condition.

### 2.3 Nutritional Quality Traits and Nutrient Use Efficiency

Wheat genetic resources are an ideal solution for addressing the issue of nutritional security in the Indian population. Malnourishment exists both in underprivileged rural populations as well as in wealthier urban populations, where anemia is a major health challenge in children and women (Müller and Krawinkel, 2005; Sethi et al., 2020). Due to its consumption by a major chunk of the Indian population, the development of iron, zinc, and protein fortified wheat is well justified as it can provide these essential micronutrients and proteins through routine edible product intake. (Borrill et al., 2014; Balk et al., 2019). The main objectives for the quality improvement are the enhancement of protein contents, bio-fortifying with essential amino acids which are basically absent in wheat, elevation in flour quality by modifying starch and glutenins, and elimination of anti-nutrient factors like phytic acid and polyphenols (Grewal and Goel, 2015; Adhikari S. et al., 2022). Although basic research on flour quality was documented, a translation aspect for wheat improvements would be fruitful. Screening of massive wheat germplasm for quality traits and their utilization for quality breeding would be an appropriate sustainable solution (Joshi et al., 2007b; Ramadas et al., 2019). However, breeding in wheat is quite difficult due to complex genetic and metabolic networks, differences in wheat plants’ micronutrient use efficiency, translocation coherence, source-sink relationship for metabolite allocation and partitioning, and genotype-dependent metabolite translocation (Ramadas et al., 2019). Hence, for efficient breeding, it is necessary to understand the genetic basis of micronutrient accumulation in grains and, accordingly, explore the conserved collection for suitable resources. Plant nutrients, including nitrogen (N), phosphorus (P_2_O_5_), and potash (K_2_O) are the major and most salient nutrients required by the plants (Zörb et al., 2018; Rajičić et al., 2019). The genetic architecture of the plants plays an important role in fertilizer uptake (Sandhu et al., 2021). Therefore, different genotypes respond differently to the amount of supplied nutrients (Mkhabela et al., 2019). Not only agronomic practices but also breeding plays an important role in improving nutrient-use efficiency. As a result, nutrient-use efficient lines/varieties can be developed by modifying root architecture, stem phenology, and leaf phenology (Dharmateja et al., 2021). The variable germplasm with miscellaneous structures, viz., deep root systems, enormous taproots, and the diverse shapes of roots previously adopted to low nutrient soil needs to be assessed under highly precise and uniform conditions. The ratio of a different nutrient may be studied for better uptake and efficiency. Using the precision nutrition platform, a large number of genotypes could be evaluated with high precision and accuracy. Overall, Indian wheat germplasm could be served for such unusual traits (Table 1), which could be useful after being incorporated into desired wheat genotypes.

**TABLE 1:** Perceptual dissemination of trait-specific Indian wheat germplasm collection.

SI no.	Important trait	Accession example	Reference
1	Drought tolerant	Safed mundri and Lal mundri and Jautri	Pal et al. (2007) and Gupta and Kant (2012)
2	High yield	Jhusia, Kishva, Churi, and Farmi	Panwar et al. (2014)
3	Drought and high biomass	Bhuri mundiya	Mehta et al. (2009)
4	Softness and good biscuit-making quality	Naphal	Ram et al. (2007) and Gupta et al. (2009)
5	Tastier chapati	Lal gehun	Gupta et al. (2009) and Mehta et al. (2009)
6	Dalia and fodder	Rata and Bhati	Gupta et al. (2009)
7	Small grains and long awns	Tank	Gupta et al. (2009) and Mehta et al. (2009)
8	Salt tolerant	Kharchia	Díaz De León et al. (2010) and Goyal et al. (2016)
9	Two forms of ear head color	Kathia	Arora and Koppar (1979)
10	Long culm	Jautri	Arora and Koppar (1979)
11	High elevation adaptation	Bhotia	Tripathi et al. (2018) and Mehta et al. (2019)
12	Valley adaptation	Chanosi	Mehta et al. (2011)
13	Drought tolerant	Dapati	Pande et al. (2016)
14	Excellent chapati quality	Daulatkhani	Mehta et al. (2011) and Mehta et al. (2019)
15	Mid-hill adaptation	Dudh gehun	Panwar et al. (2014) and Mehta et al. (2019)
16	Hailstorm tolerance	Lakha	Tripathi et al. (2018)
17	Mid- to higher-elevation adaptation	Lal mundia	Kumari et al. (2018) and Kumari et al. (2019)
18	Awnletted	Mundia	Tripathi et al. (2018) and Mehta et al. (2019)
19	Grain boldness	Thanga	Mehta et al. (2019)
20	Terminal heat tolerant	Halna	Singh et al. (2005)
21	Grain yield	Bawaji	Gami et al. (2011)
22	Non-shattering	Kankoo and Dharmauri	Panwar et al. (2014)
23	High tillering	Dharnon and Shruin	Panwar et al. (2014)
24	Long spike	Dholia and Katta	Panwar et al. (2014)

## 3 The Wealth of Indian Wheat Genetic Resources and Its Conservation Status

Archaeological and botanical evidence reveals the domestication center of einkorn (*Triticum monococcum*) and emmer (*Triticum dicoccum*) to be in the Mesopotamian crescent of the Near East at about 7500 BC (uncalibrated) and from there, it spread to the Middle East, Asia, and North Africa, and ultimately Europe, America, and South Africa (Gustafson et al., 2009). In an evolutionary context, the A genome of wheat is predominated and domesticated earliest during wheat evolution. It circumscribed to cultivate as wild einkorn. With the introduction of large-scale genomics analysis like genotype by sequencing, SNP array revealed that the origin of *T. urartu* is the closest genome for subgenome A (Maccaferri et al., 2019; Adhikari L. et al., 2022). Furthermore, with the commencement of remarkable genetic diversity losses in the pericentromeric and donating B genome by *T. speltoides* give a new tetraploid *T. turgidum*. A second hybridization event between the resulted tetraploid and third D genome donor followed by chromosome doubling has occurred to gain hexaploidy or *T. aestivum* (Gustafson et al., 2009; Maccaferri et al., 2019). Widely cultivated with dynamic adaptability, wheat can be grown at varying altitudes ranging from the sea level to 4500 m above the mean sea level (AMSL) under diverse agro-ecological conditions. Currently, it comprises several high-yielding varieties suitable for a wide range of environments, ranging from the low-humid regions of India, Nigeria, Australia, and Egypt to the highly humid regions of South America (Damania et al., 1997). Currently, the Asian continent is the leading wheat producer. For example, the total area under wheat crops is nearly 31 million ha, divided into three ecozones: the Northern Himalayan Zone, the Central Zone, and the West South Zone (Kulshrestha, 1985; Singh et al., 2007). The crop gene banks came into existence in response to the growing concern over the rapid erosion of agro-biodiversity due to the preference of superior modern cultivars over landraces and indigenous lines (Díez et al., 2018). Recognizing and deploying relevant genetic and genomic variation from wheat germplasm stored at gene banks to breeding programs is an important strategy for sustaining crop genetic improvements and conserving genetic diversity (Mir et al., 2012; Sehgal et al., 2015; Mondal et al., 2016). Recent next-generation studies have charted new approaches for eliminating redundant duplication in large gene bank collections, thus facilitating the availability of manageable collection sizes for effective molecular breeding (Mascher et al., 2019; Singh et al., 2019). Gene banks around the world maintain a huge collection of wheat germplasm (Prada, 2009). The Indian wheat genetic resources are collectively conserved in its National Gene Bank (NGB) located at the NBPGR, New Delhi.

The National Bureau of Plant Genetic Resources (NBPGR) is an official institute at the national level for the governance of plant genetic resources (PGR). Its headquarters is in New Delhi. The Indian NGB housed at ICAR-NBPGR has also been reported to store more than 31,000 wheat accessions, which include landraces, exotic lines, and indigenous collections (Tyagi, 2016). The major mandate of the institute is to intend, assemble, and coordinate exploration and collection of native and exotic plant genetic resources from extreme environments for sustainable agriculture and to introduce, exchange, and supervise intellectual property right-based quarantine of plant genetic resources.

**FIGURE 2:**
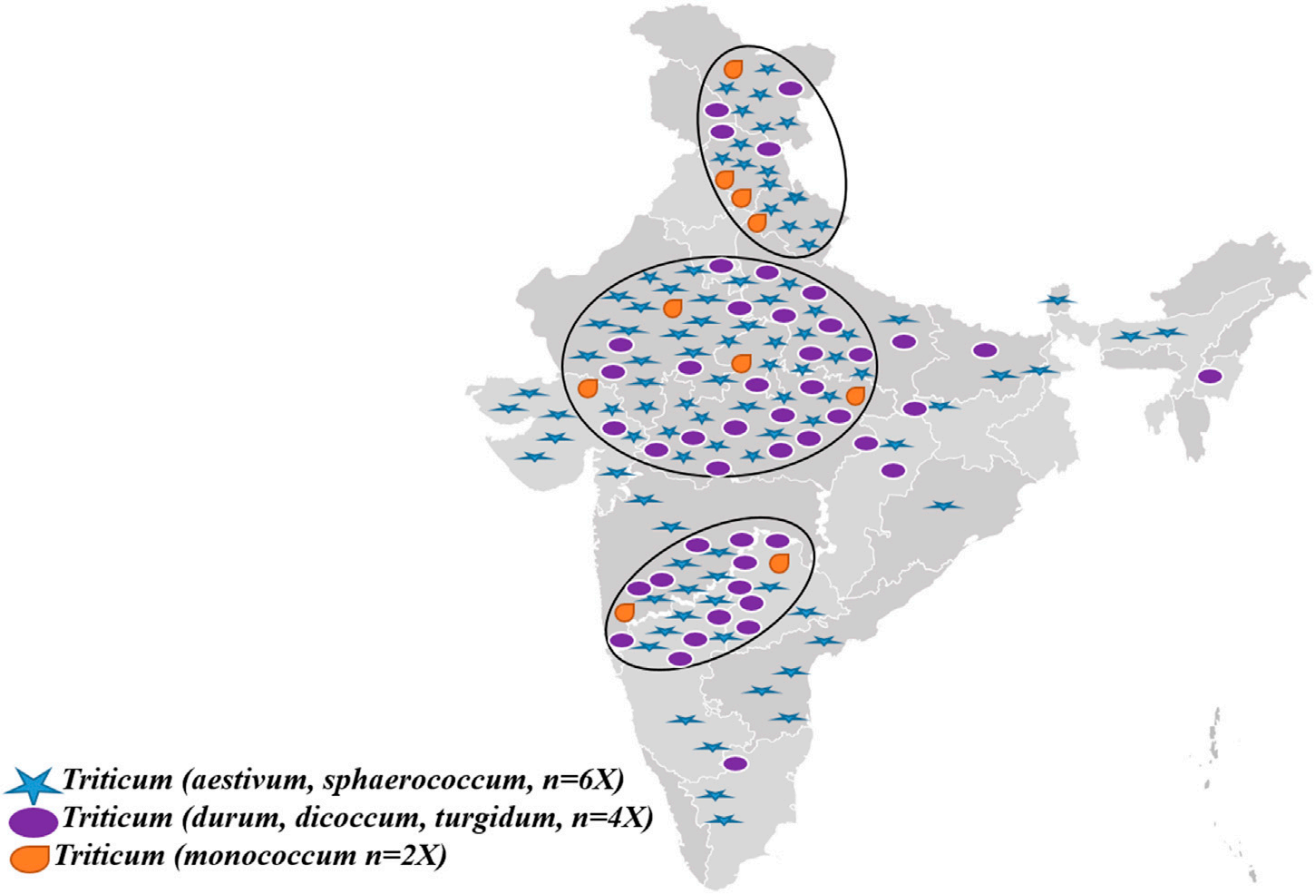
Prevalence and collection sources of diverse ploidy wheat genetic resources in India.

These are of infinite value for agriculture, food, research materials, human resources development for sustainable agricultural growth, boosting the efficient use of genetic and genomic resources of cereals, pulses, and other orphan and ornamental crops, and allied research (Singh S. et al., 2018). In addition, coordinating, capacity building in PGR management, germplasm policy access, and sharing social benefits are also pivotal. Genetic and molecular profiling of agri-horticultural crops, genetically modified plant (GMP) detection technology research, and development of information networks on plant genetic resources (Tyagi, 2016; Singh, 2018) are also mandated activities of NBPGR. Currently, the NGB of India has the largest collection of wheat in the Asian region, with around 34,000 accessions (of 51 species) in its long-term storage (data not ported in Genesys). This collection has over 18,000 indigenous and 14,000 exotic accessions (Tyagi, 2016). The wheat genetic resources are further complemented by other institutes within the National Agricultural Research System (NARS), viz., the Indian Institute of Wheat and Barley Research (IIWBR) in the Karnal district of Haryana, Punjab, Agricultural University in the Ludhiana district of Punjab, and the Indian Agricultural Research Institute (IARI), New Delhi. India’s national wheat germplasm collection is genetically rich in its species diversity and indigenous wealth. It has around 2,000 accessions belonging to the category of traditional cultivars’/landraces’/farmers’ varieties, drawn from diverse ecological zones within the country. The states of Uttarakhand, Himachal Pradesh, Uttar Pradesh, Rajasthan, Gujarat, and Madhya Pradesh are the major areas from where these indigenous resources have been collected and conserved over the past 5 decades (Figure 2). All these genotypes are treasure mines of unique genes related to several economically important traits. For example, as annexed in Table 1, drought-tolerant genotypes are Safed and Lal mundri, high yield-producing genotypes are Jhusia, Kishva, and Churi, excellent chapatti-making genotypes including Kankoo, Dharmauri, and Lal gehun, and biscuit-making quality exhibited by Mishri and Naphal. However, Bhati could be utilized as excellent fodder for livestock and Bhuri mundiya for high biomass. Additionally, augmentation efforts have been made through repatriation of Indian origin accessions from the USDA Gene Bank, Australian Grains Gene Bank, and John Innes Institute, United Kingdom. The repatriated germplasm resources comprise landraces, wild species, and relatives, which are the critical components of the wheat improvement program.

These accessions might prove very useful in the development of high-yielding and climate-resilient wheat varieties if the gene/germplasm is deployed in adapted cultivars in a planned way. However, only a small proportion of this collection has been utilized in breeding programs to date, primarily due to a lack of information about the traits and associated genes/markers in this collection. The evaluation of huge gene bank collections for the targeted traits is a costly and labor-intensive task. Although recent efforts have been made to develop core sets based on agro-morphological traits, these may not accurately represent the original collection’s diversity because agro-morphological parameters are influenced by environmental conditions (Dutta et al., 2015). Precise characterization and documentation of these valuable germplasm lines are prerequisites for germplasm utilization in breeding and genomics studies (Table 1). These germplasms have been characterized by several traits in recent years, indicating that a reasonable number of indigenous germplasm lines are tolerant to both biotic and abiotic stresses because they have co-evolved with their environments for a long time.

## 4 Identification of Trait-Specific Genetic Resources for Disease Resistance, Nutrition, and Climate Resilience

Global warming is severely affecting weather patterns, reflecting extreme heat, drought, frequent frost, and snowfall in high altitudes (IPCC, 2013). In the last few years, drought and heatwaves have become frequent in a large part of India, posing a serious threat to wheat production. During 2014–15 and 2015–16, wheat production was far below the expected target due to severe drought in various regions of the country. Soil salinity and sodicity are also anticipated to increase from the current 6.73 million ha to 20 million ha by 2050 (Sharma et al., 2012). The wheat-breeding program for abiotic stress tolerance, especially for drought and heat stresses seems to be challenging. The slow genetic progress accomplished to date is a consequence of non-adaptive genotypes with concerned environments, yield constituent compensation, the enigmatic origin of drought, and heat tolerance (Maich et al., 2007). Landraces have long served as the source of traits for local adaptation, tolerance to various stresses, yield stability, and optimum nutritional profile. Evaluation of landraces and local germplasm for finding traits pertaining to abiotic stress tolerance, and their deployment in the elite breeding lines could be the best strategy. Although, in the recent decade, scientists from NBPGR and their collaborators worldwide have made huge efforts to harness the genetic potential prevalent in wheat genetic resources (Table 2), a large proportion is still untouched in the context of trait identification and omics study.

**TABLE 2:** Recently harnessed wheat germplasm for biotic and abiotic stresses and nutrient-use efficiency to improve crop productivity.

SI no.	Evaluated germplasm	Trait	Reference
1	19,460 germplasm lines were evaluated for wheat powdery mildew	Powdery mildew resistance	Vikas et al. (2020)
2	Auspicious 47 germplasm accessions, including 15 durum species were studied for heat stress	Heat tolerance	Sareen et al. (2020)
3	169 accessions and wild relatives of wheat, viz., *Elymus* L. (8/139 acc.), *Hordeum* L. (2/4), and *Leymus* Hochst. (2/26) were assessed for cold tolerance	Cold stress tolerance	Pradheep et al. (2019)
4	287 wheat association mapping initiative (WAMI) panels used for spot blotch resistance mapping	Spot blotch resistance	Ahirwar et al. (2018)
5	Germplasm genotypes ET127225, ET127230, EC531185, ET127236, ET127267, and ET127269 exhibit a good level of drought stress tolerance	Drought tolerance	Kumar et al. (2018)
6	Accessions IC564121, IC529684, IC443669, IC443652, IC529962, IC548325, and EC178071-331 were highly resistant to spot blotch	Spot blotch	Kumari et al. (2018)
7	19,460 wheat germplasm accessions evaluated for rusts and spot blotch	Rust and spot blotch resistance	Kumar et al. (2016b)
8	The selected accessions (IC445595, IC543417, IC252650, IC310590, IC539561, IC443636, and IC75246) were evaluated and found superior for grain yield, 1,000-grain weight, and heat stress tolerance	Terminal heat tolerance	Kumari et al. (2015)
9	Out of 267, 239 accessions of *Aegilops tauschii* were resistant to stem rust	Stem rust resistance	Vikas et al. (2014)
10	Phenological and agronomical features of elite germplasm were screened against spikelet fertility	Fertility	Meena et al. (2013)
11	An inter-varietal RIL mapping population of the cross “C306” × “HUW206” was evaluated for drought stress	Drought tolerance	Kumar et al. (2012)
12	Germplasm lines for multiple disease resistance were identified	Rusts, foliar blight, and Karnal bunt	Sharma et al. (2012)
13	Germplasm lines were screened for yellow rust resistance genes (*Yr5, Yr10, Yr15*, and *Yr18*)	Stripe rust resistance	Mukhtar et al. (2015)
14	Cold and drought tolerance was observed in TW 9336, RL 111 P2, and RL 124–2 P2 along with the high grain yield and harvest index	Drought and cold stresses	Gupta et al. (2005)
15	The alleles *Barc 1*, *Barc 26*, *Barc 77*, and *Barc 147* were used to screen 41 genotypes for diversity	Genetic diversity analysis	Kumar et al. (2016a)
16	Six genotypes (IC 542394, IC 542391, IC 542416, IC 542431, IC 542426, and IC 542387) were high in Fe and Zn content	Micronutrient concentration	Krishnappa et al. (2019)
17	Drought stress tolerance was accessed in wheat germplasm	Drought tolerance	Jamla et al. (2017)
18	Six resistant landraces, viz., IC266831, IC266872, IC393109, IC392578, IC444217, and IC589276, were identified against pests	Pest, a weevil (*Sitophilus oryzae*)	Tripathi et al. (2017)
19	Multi-environmental evaluation of wheat germplasm identifies potential donors for disease resistance	Fungal resistance	Kumar et al. (2019b)
20	Exotic line characterization for disease resistance	Rusts and spot blotch	Kumar et al. (2020b)
21	Five genotypes from advanced Indian wheat breeding material were found resistant against rusts	Yellow rust and powdery mildew	Sood et al. (2020)
22	Stripe rust resistance was observed in eight genotypes including DWR 16, VL616, UP212, HD2281, HD2307, K65, Lal Bahadur, and HD2329	Stripe rust	Gupta V et al. (2018)
23	Five landraces VHC(BD)2, VHC6185, VRB-CW-2106, VHC6178, and VAH-CW 3166 revealed seedling and adult plant resistance	Stripe rust	Raghu et al. (2018)
24	IC-368665, IC-78696, IC-75352, IC-104550, IC-75354, IC-36867, IC-572071, IC-104561, 145,953, and IC-59137 exhibit QTL for stay-green trait	Terminal heat	Kumar et al. (2016c)
25	Nutrient use efficiency was observed in BW66, BW103, BW104, BW143, and BW183	Phosphorus-use efficient	Dharmateja et al. (2021)
26	Genotypes Glu3 and PBW343 + Glu acquired allele for grain protein content and test weight	Flour quality	Vishwakarma et al. (2015)
27	About 35 exotic genotypes express slow resistance to stripe rust	Stripe rust	Singh G et al. (2017)
28	Waterlogging tolerance was found in DUCULA 4, CUNDERIN, KRL 105, HD3086, RW3684, BH 1146, DBW39, 52, NW1014, NW 1067, NW 4081, PBW 621, PBW 631, PBW 590, HD 2967, HD 2997, and NW 4083	Waterlogging	Singh R. P et al. (2017) and Singh S et al. (2018)
29	IC611273, IC611071, IC75240, IC416188, IC321906, and J31-170 manifest against abiotic stress	Heat stresses	Kumar et al. (2020a)
30	About 36 wheat genotypes and three triticales were resistant against stem rust pathotype, *Ug99*	*Ug99* (stem rust)	Sharma et al. (2015)
31	Characterized wheat germplasm for puroindoline proteins (antimicrobial)	Antimicrobial properties	Chugh et al. (2015)

Hays et al. (2007) report that high temperatures during grain filling can cause a yield potential loss of up to 40% under dreadful stress. Drought is limiting wheat production in different parts of the world (Fahad et al., 2017; Abhinandan et al., 2018). Globally, about 17% of the wheat cultivated area is distressed by drought (Dai, 2013). In India, the fraction of total cultivable land affected by drought is 29%, of which 10% is under severe drought (Anonymous, 2003). Water has emerged as a limiting factor for sustained cultivation of wheat and other crops in various parts of India, even in the water-rich Indo-Gangetic Plains (Joshi et al., 2007a). Therefore, the generation of drought-tolerant varieties through breeding is essential for achieving enhanced crop productivity and food security for the hundreds of millions of people living in rural areas (Ortiz et al., 2008). Excellent drought-specific markers were identified to determine tolerance against droughts such as *Dreb* and *Fehw3* (Rasheed et al., 2016). Consequently, the existence or absence of *Dreb* and *Fehw* markers can be analyzed in any promising germplasm. Several drought-tolerant lines have been identified in India (Kumar et al., 2018), which can be used for favorable allele mining. The identification of novel genetic loci for the improvement of drought tolerance can be achieved by GWAS or QTL mapping using germplasm lines (Zeng et al., 2014; Sukumaran et al., 2018) (Table 2).

## 5 Availability/Discovery/Development of Genomics Resources

Over the past decade, there has been a substantial advancement in the development of genomic tools and techniques in wheat (Alaux et al., 2018; Purugganan and Jackson, 2021). The wheat gene pool possesses a tremendous amount of genetic variability for a trait of interest. Several high-density genetic and physical maps of wheat have been developed (Chao et al., 2007). The release of the gold standard reference genome assembly of wheat into the public domain will expedite the use of genomic resources in breeding (IWGSC, 2018). Moreover, high-throughput genotyping tools such as SNP arrays and GBS platforms have also been developed. In recent years, there has been an outburst of innovations in the field of “genomics” which can be employed for the identification of genes or genomic regions for useful traits from a large set of germplasm collections conserved in gene banks (Crossa et al., 2016; He and Li, 2020). The important ones to mention are high-throughput genotyping assays, whole-genome sequencing (WGS), GWAS, and genomic selection (GS) (Muleta et al., 2017). Of these, GS is of special interest and has emerged as a promising approach for genetic improvement of complex traits (Ali and Borrill, 2020; He and Li, 2020). GS could be used for large plant breeding populations with genome-wide molecular markers to predict the total genetic value for complex or economically important traits such as yield. The key conceptual difference between conventional breeding and genomic selection approaches is that in the former, selections of candidate varieties are based on the observed phenotypic performance, whereas, in the latter, selections are based on the genetic makeup and genotype × environment interaction (Crossa et al., 2016; He and Li, 2020). A robust theoretical and experiential report suggests that GS methods can predict performance with adequate accuracy to allow selection based on molecular markers alone (Ali and Borrill, 2020). Furthermore, GS is a promising approach for accelerating the rate of genetic gain in plant-breeding programs by enabling selection for complex traits (like yield under heat stress) early in the breeding cycle and therefore reducing the cycle time, which increases the annual gain. Genomic selection has a potential breeding strategy to map numerous genetic loci for diverse traits of interest. Various research groups started working on Indian wheat and associated germplasm genotype for crop improvement against biotic resistance (Juliana et al., 2021; Budhlakoti et al., 2022). But still, more accurate prediction from a large genotype reservoir of Indian wheat germplasm is necessary for germplasm-assisted crop improvements for abiotic and quality-related traits.

**FIGURE 3:**
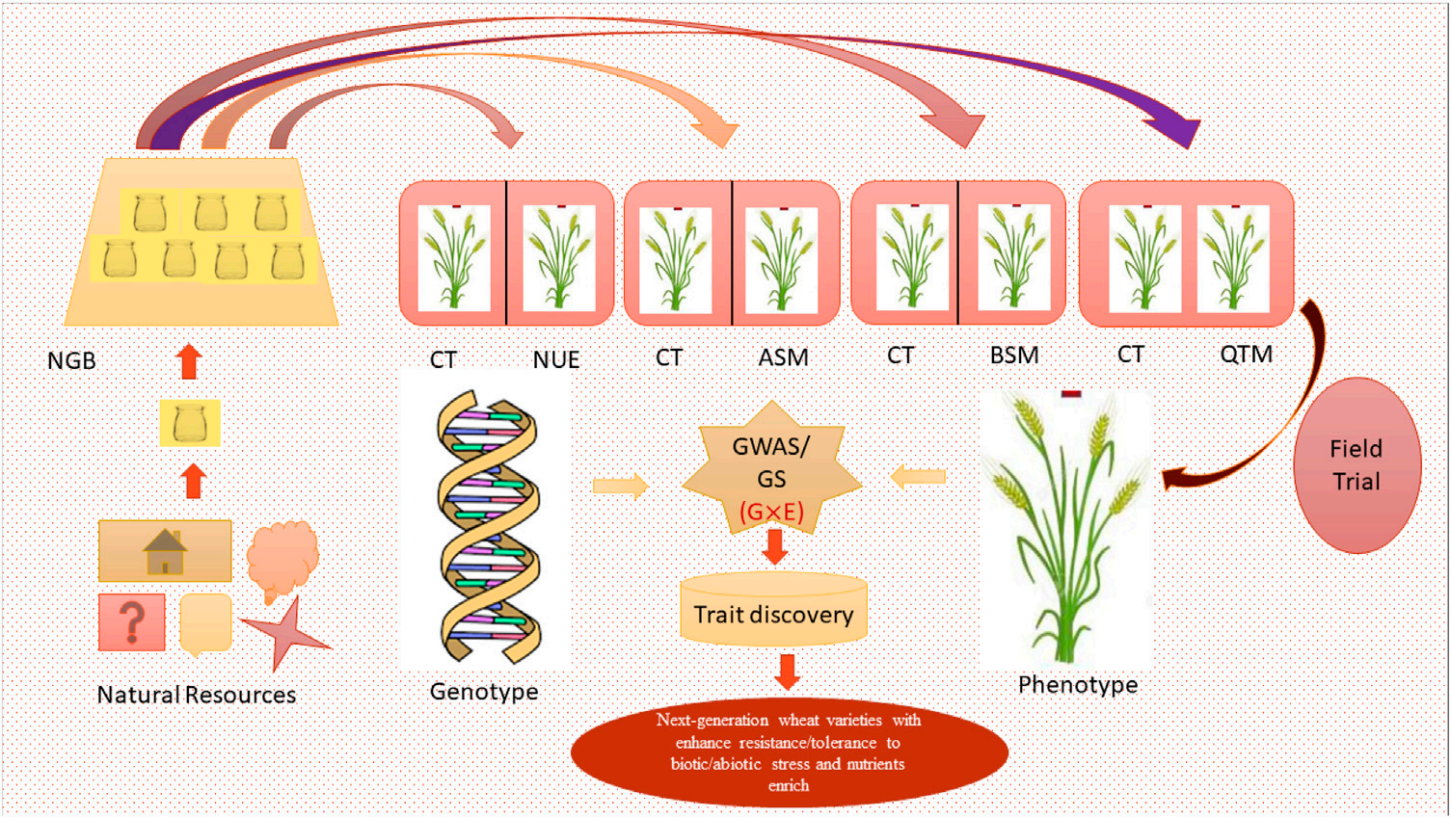
Roadmap for germplasm assessment and trait discovery using integrated analysis of the genotype and phenotype (NGB—National Gene Bank; CT—control; NUE—nutrient-use efficiency; ASM—abiotic stress measurement; BSM—biotic stress measurement; QTM—quality trait measurement; GWAS/GS—genome-wide association study/genomic selection).

In GS, genome-wide molecular markers are used to predict total breeding values called genomic estimated breeding value (GEBV) and make selections of individuals or breeding lines before phenotyping (Larkin et al., 2019; Kumar et al., 2021). This approach has several advantages, especially for 1) making selections before phenotypic evaluation, which reduces the time needed to make selections and 2) increasing the size of breeding populations since genotyping of a large number of lines can be carried out at a lower cost than phenotypic evaluation (Crossa et al., 2016; Muleta et al., 2017; He and Li, 2020). This component aims to develop genomic selection for yield-related traits to accelerate genetic gain. Genomic selection approaches have been proven to be effective for complex or economically important traits such as yield, using an elite set of lines, including germplasm (Roy et al., 2021). From our perspective, there is little information available about Indian wheat germplasm, and a short table was prepared (Table 3) in the context of genes, transcripts, and QTL identified so far. In addition to the huge sustaining potential (Table 1), the Indian germplasm lines will be used to develop a prediction model using the existing genotyping and multi-location phenotyping data (Figure 3). The focus of this review would be to envisage candidate genotypes preserved in the national gene bank, which can be produced in abundance under varying climatic conditions. To the best of our knowledge, this would be the first time that genomics- and physiology-based hypothetical networks would be used to maximize the value of wheat germplasm in India. The collection of representative lines in this study will generate a public resource of elite germplasm lines with well-characterized phenotypic and genotypic information, along with seeds and their genetic constitutions. This resource would lead to determining the optimized configuration of wheat-breeding systems to support coming generations.

**TABLE 3:** Identified candidate gene/transcripts, QTL, and MTA, etc., from Indian wheat germplasm.

Candidate gene, QTL, transcript, and MTA	Desired trait	Reference
*Pina and Pinb* gene	Grain hardness	Chugh et al. (2015) and Kumar R. et al. (2015)
*tsn 1* gene	Tan spot resistance	Phuke et al. (2020)
*APR* gene detection	Leaf rust	Kumar et al. (2019a)
*pgd3* gene	Heat stress	Rangan et al. (2020)
(TA 5088 and TA 5638) Alien chromosome	Drought tolerance	Djanaguiraman et al. (2019)
SNPs	Spot blotch resistance	Ahirwar et al. (2018)
Glu-B1	Protein quality	Routray et al. (2007)
Putative *Pm3c*	Powdery mildew	Basandrai et al. (2016)
Alleles 12*, 12.1*, 12.1, 12.2, and 12.3	Glu-D1 locus	Goel et al. (2018)
*vrn*-B3, Vrn-A1c, GluB3i, GluB3g, and GluA3b	Vernalization and glutenin	Sehgal et al. (2015)
MTAs (2AS, 1BS, 6BS, and 7BL) significant (2NS/2AS translocation)	Head blast resistance	He et al. (2021)
MTAs on 2A, 3A, 1B, 2B, 3B, 4B, 5B, 6B, 2D, and 3D	Stripe rust resistance	Pradhan et al. (2020)
Multi-trait SNPs on Chr2BS, Chr1Ds, and Chr2DS	Agronomic traits	Kumar et al. (2020a)

## 6 Future Prospects of Harnessing the Genetic Potential of Wheat Germplasm Using Genomic Approaches

The outcome of the Indian wheat germplasm genomics initiative would be a comprehensive pipeline connecting germplasm evaluation and genomic information, which could be used to accelerate the utilization of indigenous wheat germplasm in the national breeding programs for the improvement of biotic, abiotic, and quality traits (Figure 3). The following envisaged expected output could be: 1) a detailed insight into the extent and pattern of quality traits in the indigenous wheat collection, 2) wheat germplasm and genomic resource database containing phenotypic evaluation data and associated genomic information in the form of SNP markers for large-scale genotyping applications, 3) molecular tags such as markers, genes, and haplotypes associated with important agro-morphological, yield, and grain quality associated traits, 4) novel gene/markers conferring resistance to important wheat diseases (rusts, powdery mildew, and spot blotch, etc.) and tolerance to environmental stresses (heat, drought, and salinity), 5) elite germplasm/accession/genetic stocks based on extensive phenotyping and genomics-based analysis, 6) stable and cross-validated genomic prediction model to calculate the genomic-estimated breeding value for faster genetic gain in elite and pre-breeding lines for various traits (heat, drought, nitrogen use efficiency, rusts, spot blotch, and yield, etc.), 7) integration of physiological traits into the national wheat-breeding program to develop high carbon-capturing pre-breeding lines or candidate varieties, and 8) rematriation of old landraces of wheat to evaluate them at their native place to know their adaptive functionality. Wheat genetic resources include extant cultivars, obsolete cultivars, parental lines, advanced breeding material, mapping populations, and explored germplasm lines. Globally, there is a huge reserve of conserved wheat genetic resources, though much of it remains unexplored for trait-specific information. Genetic data on traits and their association with suitable markers will facilitate the use of wider variability in crop improvement. In this project, such an effort has been put forth to strengthen the Indian wheat-breeding program. The pre-breeding lines generated in the project will have enhanced climate resilience and combining both abiotic and biotic stress tolerance and maximized yield potential. This work would also set a precedent for further enrichment of the national wheat collection with wild and weedy relatives of wheat (wild Triticaceae such as species of *Aegilops*, *Elymus*, and *Eremopyrum*), distributed primarily in the western and north-western Himalayas, for use in future programs on climate resilience. It would also serve as a reference for identifying the required areas for exploration and collection of *Triticum* species based on the gaps identified in the gene bank holdings, especially for trait-specific and unique germplasm accessions.
